# Next Generation—Sébastien R. Mouchet

**DOI:** 10.3390/biomimetics3020010

**Published:** 2018-05-09

**Authors:** Lidia Garcia-Campmany

**Affiliations:** *Biomimetics* Editorial Office, MDPI AG, St. Alban-Anlage 66, 4052 Basel, Switzerland; lidia.garcia@mdpi.com

## Abstract

Next Generation is a series of interviews with the awardees of the *Biomimetics* Travel Awards aimed at supporting early-career researchers and helping them promote their work. Sébastien R. Mouchet is a postdoctoral fellow in the Natural Photonics group led by Prof. Pete Vukusic at the University of Exeter, UK, working in collaboration with his former Ph.D. supervisor, Prof. Olivier Deparis, at the University of Namur, Belgium. His research focuses on fluorescence emission and coloration changes in photonic structures of insects induced by contact with fluids aiming to develop bioinspired technological solutions for chemical sensing and biosensing.


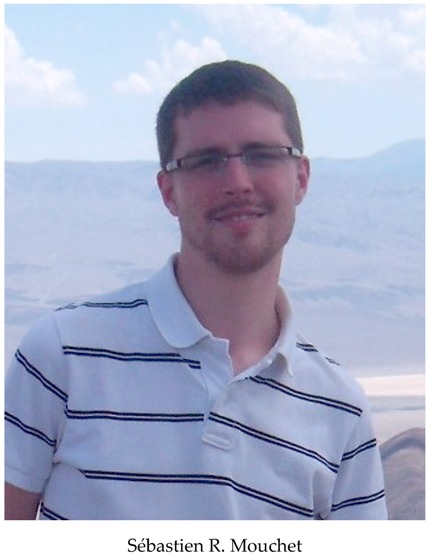


## How did you first become interested in the physics of structural colors?

As a Physics undergrad student at the University of Namur, Belgium, I followed the lectures of the late Prof. Jean-Pol Vigneron who studied the physical origins of natural organisms’ visual appearances. He was gifted in the way in which he talked about his research and very soon, I became passionate about the topic. During my M.S. degree in Physics, I had the opportunity to be introduced in this field through an internship in Prof. Doekele Stavenga’s group at the Zernike Institute for Advanced Materials of the University of Groningen, The Netherlands. I was hooked. In addition to the beauty of the investigated samples—optical micrographs are usually stunning—such studies comprise key challenges in physics and biology with application developments that are sometimes possible through a bioinspired approach. Back from The Netherlands, I decided to complete an M.S. thesis on structural colors in iridescent beetles in Prof. Vigneron’s group. Later on, I continued along this line of research with a Ph.D. thesis.

## What aspects of your research you enjoy the most?

I do enjoy the multidisciplinary aspects of my research which involves many techniques that I have learned and requires the interaction with experts from various fields. Regarding the physical aspects of my research, both experimental and numerical investigations usually need to be performed. It is always very satisfying when experimental and numerical results match since it usually means that the photonic behavior has been understood.

## Can you tell us about an unexpected finding that led you to a major discovery? 

I am unsure whether this may be called a “major discovery” per se, but a few years ago, during the final year of my Ph.D., while investigating the liquid-induced color changes of the male *Hoplia coerulea* beetle (from shiny blue to green) by optical microscopy [1], Dr. Eloise Van Hooijdonk, at that time a postdoctoral researcher at the University of Namur, stopped by the microscopy room on her way to the coffee room. We discussed the measurements I took that morning and she confessed to me that when she studied the fluorescence emission properties of this beetle from a numerical point of view during her Ph.D., she did not perform any experimental observations. I therefore promised her that by lunchtime I would produce a beautiful image of the beetle’s wing for her using fluorescence microscopy. I did so, and the image was eventually nominated “Image of the Week” by the Optical Society (OSA)’s *Optics & Photonics News* magazine [2]. As I was investigating the liquid-induced color changes of this beetle when I took this image, we became both curious to observe the liquid-induced changes of fluorescence emission from this beetle. The result was quite counterintuitive and trying to explain these observations I noticed that changes induced by liquids in optical properties of insect photonic structures other than color are generally underinvestigated. A few months later, I secured a research fellowship related to that subject. It allowed me to carry out my first postdoctoral project at the University of Exeter, UK. In addition, the results were published in the *Proceedings of the Royal Society B* [3]. I believe this anecdote should encourage early-career researchers to perform observations that are driven by their own curiosity and to follow their scientific instinct, even if this leads them to perform experiments that are not really related to their own research project. Eventually, if they think their ideas are grantable, they should not hesitate to write their own proposal. You are never too young for it!

## What was the importance for you of winning the *Biomimetics* Travel Award?

With the *Biomimetics* Travel Award, I could present the results of my research at the Living Light conference (http://www.livinglight-conference.org/) held in Cambridge, UK, on 11–14 April 2018. This conference is a biennial interdisciplinary event dedicated to natural photonics and bioinspired applications. It gathers biologists, physicists, engineers, and chemists from all over the world. I made two contributions: an oral presentation on liquid-induced fluorescence changes in beetles’ photonic structures [4] and a poster presentation on structural coloration in Diptera [5]. Presenting my work at this conference was very important for my current project, my career, and both research groups in Namur and Exeter because of the significance of our results from both the biological and biomimetic points of view. In addition to being an effective dissemination forum, this meeting provided me with unique opportunities to exchange ideas with leading researchers and to learn about the latest developments in the fields of natural photonics and biologically inspired optics.

## What are your plans for the future?

I have recently been awarded a fellowship funded by the Belgian National Fund for Scientific Research (F.R.S.-FNRS) and started out this new postdoctoral position at the University of Exeter. My current research project is a joint venture between the groups lead by Prof. Pete Vukusic and my former Ph.D. supervisor, Prof. Olivier Deparis. My current project focuses more generally on the optical response of natural organisms’ photonic structures to ultraviolet light. My future plans, of course, are made around this project and will require me to learn new techniques and skills. 

## What are the challenges and needs of early-career researchers?

The main challenges of early-career researchers are probably related to job security and the two-body problem. A research position is usually a (very) short-term position. It might be challenging for early-career researchers to focus on their research when looking for the next position or applying for fellowships. It might be even more challenging if they need to take into account their significant other’s career. Solving exactly such challenges is probably hard and requires trade-offs and luck, I guess, in order to find the right balance.

## What advice would you give to someone who is starting out on their Ph.D.?

I would recommend Ph.D. students to follow their passion, although it is not always easy, and to talk to people not only in their own lab but also from other labs at their University, or at conferences in order to exchange information about their subject and get other points of view on their own research. It is also important not to become discouraged. “Keep calm and carry on.” The results of a Ph.D. thesis may not be the ones expected at the beginning. That does not mean they are worthless. If performed with rigor, they will always fit nicely in the manuscript of a Ph.D. thesis. When things seem to be going wrong, there is always someone around to help you and cheer you up.

## What do you like doing out of the lab?

Outside the lab, I do enjoy sharing my knowledge with others and talking about science in general and my research topics (e.g., in the context of seminars for students and pupils, outreach events, or pop-science articles). I also like teaching through lectures, tutorials, and demonstrations. Besides doing research and teaching, I play soccer and futsal (i.e., an indoor five-a-side version of soccer). I also like walking in nature, studying history, as well as discovering new countries and cultures.
